# The Way of Water: Unravelling White Spot Syndrome Virus (WSSV) Transmission Dynamics in *Litopenaeus vannamei* Shrimp

**DOI:** 10.3390/v15091824

**Published:** 2023-08-28

**Authors:** Natasja Cox, Evelien De Swaef, Mathias Corteel, Wim Van Den Broeck, Peter Bossier, João J. Dantas-Lima, Hans J. Nauwynck

**Affiliations:** 1 IMAQUA, 9080 Lochristi, Belgium; evelien.swaef@imaqua.eu (E.D.S.); mathias.corteel@imaqua.eu (M.C.); joao.lima@imaqua.eu (J.J.D.-L.); 2 Laboratory of Virology, Department of Translational Physiology, Infectiology and Public Health, Faculty of Veterinary Medicine, Ghent University, 9820 Merelbeke, Belgium; hans.nauwynck@ugent.be; 3 Department of Morphology, Medical Imaging, Orthopedics, Physiotherapy and Nutrition, Faculty of Veterinary Medicine, Ghent University, 9820 Merelbeke, Belgium; wim.vandenbroeck@ugent.be; 4 Laboratory of Aquaculture & Artemia Reference Center, Department of Animal Sciences and Aquatic Ecology, Faculty of Bioscience Engineering, Ghent University, 9000 Ghent, Belgium; peter.bossier@ugent.be

**Keywords:** white spot syndrome virus, *Litopenaeus vannamei*, natural history of disease, transmission dynamics, water-borne disease transmission

## Abstract

White spot disease (WSD) is a severe viral threat to the global shrimp aquaculture industry. However, little is known about white spot syndrome virus (WSSV) transmission dynamics. Our aim was to elucidate this in *Litopenaeus vannamei* using peroral *in vivo* WSSV challenge experiments. We demonstrated that WSD progression was rapid and irreversible, leading to death within 78 h. Viral DNA shedding was detected within 6 h of disease onset. This shedding intensified over time, reaching a peak within 12 h of the time of death. Isolating shrimp (clinically healthy and diseased) from infected populations at different time points post-inoculation showed that host-to-host WSSV transmission was occurring around the time of death. Exposing sentinels to environmental components (i.e., water, feces, molts) collected from tanks housing WSSV-infected shrimp resulted in a significantly (*p*-value < 0.05) increased infection risk after exposure to water (1.0) compared to the risk of infection after exposure to feces (0.2) or molts (0.0). Furthermore, ingestion of WSSV-infected tissues (cannibalism) did not cause a significantly higher number of WSD cases compared to immersion in water in which the same degree of cannibalism had taken place.

## 1. Introduction

For 30 years, white spot disease (WSD) has been the most widespread and lethal disease in shrimp aquaculture worldwide [1,2,3,4]. The etiological agent, white spot syndrome virus (WSSV), is a large, rod-shaped, double-stranded DNA virus. It belongs to the genus *Whispovirus* of the family *Nimaviridae*. The virus is highly virulent in commercially important species of penaeid shrimp, such as *Litopenaeus vannamei*, and can cause a cumulative mortality up to 100% within 3 to 10 days in grow-out ponds [4,5,6,7,8,9]. Although some research about immune system activation, vaccinations, RNAi, and the application of herbal extracts has shown promising results, to date no prophylactic or curative treatments for WSD are known [10]. Hence, most control measures and husbandry practices in the field aim to prevent disease outbreaks. To develop effective mitigation strategies, a better understanding of the dynamics of WSSV epidemics and transmission is still needed [11]. Unfortunately, studies that have examined the dynamics of WSSV transmission in *L. vannamei* are scarce, as reviewed by Desrina et al. [11].

Three main routes of WSSV transmission have been reported: (1) a vertical transovarial transmission from brooders to progeny [4,10,12], (2) horizontal transmission through ingestion of WSSV-infected carcasses [13,14], and (3) horizontal transmission by exposure to water-borne WSSV [15,16]. In *L. vannamei*, horizontal transmission through consumption of infected tissue, by cannibalism or predation, is often considered to be the most effective path of infection compared to exposure to water containing WSSV virions [4,10,17]. This idea was supported by the work of Soto and Lotz [17], who performed the earliest known study on WSSV transmission dynamics in *L. vannamei*. Nevertheless, a more recent study contested these findings, suggesting that direct contact transmission, for which cannibalism was considered a co-factor, was of minor importance for *L. vannamei* compared to indirect environmental transmission [15]. Therefore, the question was raised why the results from these studies were contradictory.

Viral diseases in aquaculture are the result of the interplay between pathogen, host, and environment. The transmission dynamics of a specific WSSV strain in an animal population depends on the virulence of that WSSV strain, the density of the susceptible hosts, the individual host defense status, and recovery rates [18]. To accurately characterize these dynamics, however, it is necessary to first understand the time course of WSD in an individual host. This can be examined by analyzing the natural history of disease, which is typically conducted through longitudinal studies that follow a group of infected individuals over time and monitor health outcomes [19,20]. Though many studies have been reported on infectivity [21,22,23,24], pathogenicity [5,25,26,27,28,29], and virulence of different WSSV strains [30,31,32,33,34,35,36], these concepts do not fully describe the progress of a disease process in an individual host over time from the asymptomatic stage to its cessation as recovery, disability, or death [19,37]. A natural history of disease study as such has not been reported for WSD in *L. vannamei*; this is unfortunate, because the data from such a study could be used to analyze the disease pattern and ultimately characterize the epidemic pattern of spread [38,39]. Moreover, studying the epidemiology of an infectious disease such as WSD can potentially reveal the primary transmission dynamics, as it did for AIDS and COVID-19 in humans [39,40]. Furthermore, it can aid in the determination of the point at which an individual case can become a risk factor for disease in other individuals [38], or in other words when host-to-host transmission occurs, and which risk factors are potentially influencing these dynamics [19].

In this respect, the aim of this study was to use in vivo infection modelling as a tool to analyze the dynamics of the horizontal transmission of WSD in *L. vannamei*, and to evaluate the importance of specific environmental components that might be involved in this process. First, we performed a natural history of disease study in an individual infection model using the WSSV Thai-1 strain, which has been extensively researched in *L. vannamei* [22,35,36,41,42,43,44]. Second, we developed a reproducible experimental infection model in which shrimp were housed in group. Third, this model was then used for an observational epidemiological study to identify the characteristics of an epidemic caused by Thai-1. Finally, we employed this model to investigate the role of specific environmental components (i.e., molts, feces, water) in WSSV transmission dynamics.

## 2. Materials and Methods

### 2.1. Experimental Animals and Rearing Conditions

Specific-pathogen-free (SPF) *L. vannamei* were imported as postlarvae (PLs) from Global Blue Technologies (Rockport, TX, USA). These shrimp were certified as SPF for WSSV, infectious hypodermal and haematopoietic virus (IHHNV), Taura syndrome virus (TSV), yellow-head virus (YHV), infectious myonecrosis virus (IMNV), *Enterocytozoon hepatopenaei* (EHP), necrotizing hepatopancreatitis bacteria (NHP-B), covert mortality nodavirus (CMNV), monodon baculovirus (MBV), hepatopancreatic parvovirus (HPV), acute hepatopancreas necrosis disease-causing *Vibrio* sp. (AHPND), baculovirus penaei (BP), and *Penaeus vannamei* nodavirus (PvNV). Upon arrival, PLs were transported to IMAQUA, Aquaculture Immunology Technologies (Lochristi, Belgium), and reared in a recirculating aquaculture system (RAS) equipped with 470 L tanks containing artificial seawater at a salinity of 20 ppt. They were raised on an artificial diet (MeM, Bernaqua, Belgium) and then weaned with diets formulated and produced at IMAQUA [45]. Feed was automatically distributed 6 to 12 times a day using a custom-built programmable belt feeder.

### 2.2. Experimental Conditions

For the experiments, shrimp were randomly selected from the rearing tanks and transferred to the disease challenge facility, where they were housed either in 10-L or 290-L challenge tanks and acclimatized for 3 days prior to the start of the trials. During the infection trials, shrimp were fed at a fixed feeding rate set at a level of 6.5% of their body weight. Water filtration was performed by biological and mechanical filters installed in each 290-L tank and by biological filters in each 10-L tank. Ammonia, NO_2_, and KH were tested daily using test kits (JBL, Neuhofen, Germany). The total ammonia nitrogen (TAN) levels were between 0 and 5 mg L^–1^ and the nitrite levels were between 0 and 1 mg L^–1^. The water temperature was kept constant at 27 °C ± 1 °C by means of an automatic temperature control system, and the photoperiod was 12 h light/12 h dark. Dissolved oxygen was maintained above 4 mg L^−1^ and pH ranged between 7.8 and 8.5. Temperature, dissolved oxygen, and pH were monitored with a multimeter (WTW multi 3620 IDS, WTW, Weilheim, Germany). Salinity was maintained at 20 ppt and measured with a digital refractometer (MA887, Milwaukee, Rocky Mount, NC, USA).

### 2.3. WSSV Stock and Peroral Inoculation

The WSSV Thai-1 strain was used in this study [46]. A virus stock is kept frozen at −70 °C at the facilities of IMAQUA. This strain was previously isolated in Thailand from naturally infected *Penaeus monodon* and passaged once in crayfish *Pacifastacus leniusculus* [47]. The infectivity titer of the viral suspension was determined according to a previous study [46], and the suspension was used to infect shrimp intramuscularly. The resulting infected shrimp carcasses were used to prepare WSSV-positive tissue inoculum (stock 1 and stock 2) and the infectivity titers were determined in triplicate [46,48]. Carcasses from WSSV-negative shrimp were used to prepare one stock of WSSV-negative inoculum (mock stock) [22,45]. During the peroral WSSV challenges, these tissue inocula were used to feed the shrimp naturally following the procedure described by Thuong at al. [22].

### 2.4. In Vivo WSSV Challenges

#### 2.4.1. Characterization of WSD Progression

**Figure 1:**
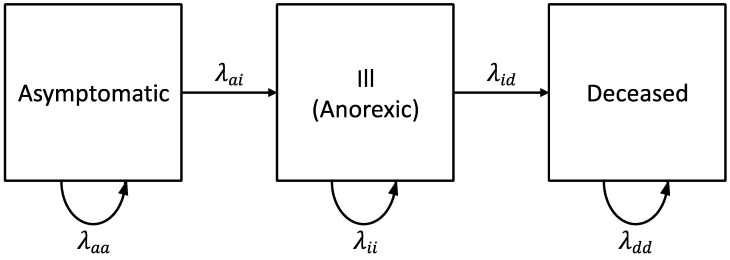
State-transition diagram for a unidirectional illness-death model. The model consists of three disease states: asymptomatic (a), ill (i), and deceased (d). Variable names adjacent to the solid arrows are transition rates (*λ*_aa_, *λ*_ai_, *λ*_ii_, *λ*_id_, *λ*_dd_). This model is said to be “progressive” because transitions are irreversible (i.e., unidirectional). The curved arrows indicate that individuals can remain in a particular state over time (adapted from [48]).

Experiment 1a: we investigated the natural course of WSD caused by WSSV Thai-1 in individually housed *L. vannamei* shrimp. The experiment was repeated four times with different batches of PLs with average weights ranging from 1.2 to 2 g. For each replicate, twenty-five shrimp were individually housed in 10-L tanks. Fifteen individuals were challenged with WSSV-positive tissue inoculum (stock 1 = 10^7.7^ SID_50_ g^−1^). Ten shrimp were given WSSV-negative tissue to serve as a negative control group (NCTRL) that should not show any mortality during the experiment, since this could indicate other causes of death. During the experiment, the occurrence of anorexia, a key symptom for WSD, was recorded and used in the initial diagnosis of suspected clinical WSD. Additionally, mortalities were registered, and dead and moribund shrimp were collected twice daily for qPCR to confirm WSSV as cause of death. The experiment was terminated when no mortalities were observed for 48 h WSD was conceptualized as consisting of three health states: asymptomatic (a), ill (i), and deceased (d). Figure 1 shows five possible state-transitions (a → a, a → i, i → i, i → d, d → d). The transition rates (*λ*) (or the probabilities per unit of time that a state transition would occur within a time interval) were calculated using the empirical data collected during the experiment:

$$\lambda_{s}(t)=\frac{n_{s}(t)}{{n\;}_{tot}},$$

where the transition rate $\lambda_{s}(t)$ is the probability that a shrimp has made a state-transition $s,\;s\;\in \;\left\{aa,ai,ii,id,dd\right\}\;$ at time interval t, $n_{s}(t)$ is the number of shrimp found in health state s at time t, and $n_{tot}$ is the total number of shrimp in the experimental population under consideration.

Experiment 1b: to detect WSSV DNA shedding by the individual animals into the rearing water, forty PLs with an average weight of 2.3 g were challenged with the same WSSV-positive tissue inoculum (stock 1 = 10^7.7^ SID_50_ g^−1^) in a separate experiment. Ten shrimp were given WSSV-negative tissue as a negative control group (NCTRL). Immediately after the inoculation, the shrimp were transferred from the 10-L WSSV-inoculation tanks to newly set up 10-L tanks that were WSSV-free to reduce the chances that WSSV DNA from the inoculum would contaminate the water samples taken during the experiment. Shrimp were housed individually in the WSSV-free tanks. The incidence of anorexia was recorded in addition to the mortalities. Dead and moribund shrimp, as well as water samples, were collected twice daily for qPCR to confirm dead by WSSV. The experiment was terminated after 48 h without mortalities. All surviving shrimp were euthanized. All carcasses and water samples collected during the challenge and at the conclusion were stored at −70 °C until qPCR analysis was performed in duplicate. *Vp19* copy numbers per mL water were calculated:

$$Vp19\;copy\;numbers/mL=\frac{\left(Vp19\;copy\;numbers\;per\;µL\;of\;extracted\;total\;DNA)\times (volume\;of\;extracted\;total\;DNA\right)}{volume\;of\;water\;used\;to\;extract\;the\;total\;DNA(mL)}$$

#### 2.4.2. WSSV Infection in Relation to Population Density

**Figure 2:**
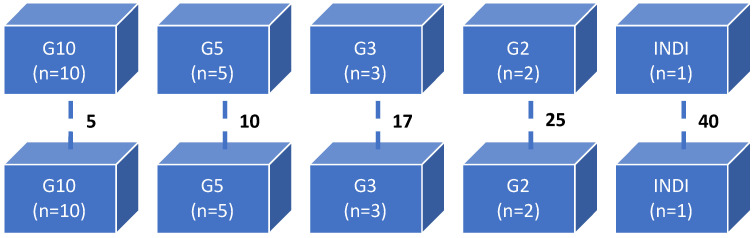
A schematic diagram showing the design of experiment 2: WSSV infection in relation to population density. The numbers next to the dashed line refer to the number of replicate 10-L tanks. Shrimp from G10, G5, G3, and G2 were housed with 10, 5, 3, and 2 shrimp per tank in 5, 10, 17, and 25 replicate 10-L tanks, respectively.

Experiment 2: the experimental design is illustrated in Figure 2 The probability of infection in populations of a certain density was determined by randomly dividing PLs with an average weight of 3.6 g into five groups: G10, G5, G3, G2, and INDI. Shrimp from INDI (*n* = 40) were housed individually. Shrimp from G10, G5, G3, and G2 were housed with 10, 5, 3, and 2 shrimp per tank in 5, 10, 17, and 25 replicate 10-L tanks, respectively. They were inoculated with WSSV-positive tissue (stock 1 = 10^7.7^ SID_50_ g^−1^). Five shrimp were housed individually and inoculated with WSSV-negative tissue, serving as a negative control group (NCTRL). Dead and moribund shrimp were collected twice a day for qPCR to confirm WSSV as cause of death. The experiment was terminated when no mortalities were recorded for 48 h. All surviving shrimp were euthanized. All carcasses, collected during the challenge and at the end of the experiment, were stored at −70 °C until qPCR analysis. The probability of infection in a population of a certain density was calculated as:

(1) $$P_{i}=\frac{n_{i}}{n_{tot}},$$

where $P_{i}$ is the probability that a population of a certain density is infected, $n_{i}$ is the number of infected populations of a certain density, and $n_{tot}$ is the total number of populations of a certain density in the experiment.

#### 2.4.3. The Effect of Isolation on WSSV Epidemic Dynamics

**Figure 3:**
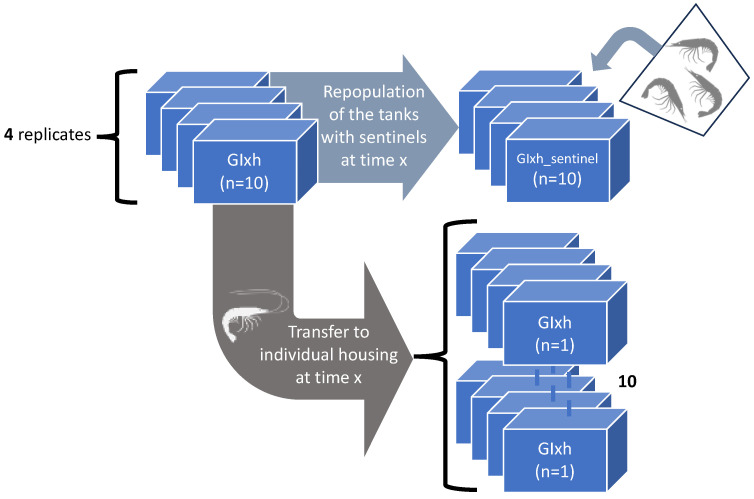
A schematic diagram showing the design of experiment 3: the effect of isolation on WSSV epidemic dynamics. The letter ‘x’ refers to the time point (5, 30, 48, 72 hpi) at which the first groups of shrimp were transferred to individual housing and the vacated tanks were repopulated with sentinel shrimp.

Experiment 3: the experimental design is illustrated in Figure 3. The WSD epidemic patterns of spread were identified as follows: PLs with an average weight of 2.2 g were randomly divided into seven groups (GI5h, GI30h, GI48h, GI72h, G∞, INDI) with 10 shrimp per group. Groups GI5h, GI30h, GI48h, GI72h, and G∞ were each housed in 4 replicates of 10 shrimp per 10-L tank. The group INDI consisted of 40 shrimp individually housed in 10-L tanks. As in 2.4.1, the shrimp from INDI, GI5h, GI30h, GI48h, GI72h, and G∞ were challenged with WSSV-positive tissue (stock 1 = 10^7.7^ SID_50_ g^−1^). Five control shrimp were housed individually and inoculated with WSSV-negative tissue to serve as a negative control group (NCTRL). Five hours after the start of the inoculation procedure, when all the WSSV-infected tissue had been consumed, the animals of the GI5h treatment were transferred and housed individually in forty newly set up 10-L tanks. After removal of these animals, four groups of ten sentinel SPF shrimp were placed into each GI5h 10-L group tank to detect the presence of infectious WSSV virus in the tank environment. In the following days, at 30 h, 48 h, and 72 h post-inoculation (hpi), the shrimp from groups GI30h, GI48h, and GI72h were transferred and housed individually in new 10 L tanks. After removal of the inoculated animals, the tanks were also repopulated with ten sentinel animals per tank. Group G∞ remained in their group housing throughout the entire experiment. During the experiment, dead and moribund shrimp were collected twice a day for qPCR. The experiment was terminated when no mortalities were recorded for 48 h. All surviving shrimp were euthanized. All carcasses, collected during the challenge and at the conclusion, were stored at −70 °C until qPCR analysis to confirm death by WSSV. Based on the empirical data, the basic reproduction number *R_0_* was calculated:

(2) $$R_{0}=\frac{n_{i,2}}{n_{i,1}},$$

where $R_{0}$ is the basic reproduction number, $n_{i,2}$ is the expected number of secondary cases (shrimp infected through host-to-host WSSV transmission), and $n_{i,1}$ is the expected number of primary cases (shrimp infected by feeding on the WSSV-positive tissue inoculum).

#### 2.4.4. Role of Molted Cuticles, Feces, and Rearing Water in WSSV Transmission Dynamics

**Figure 4:**
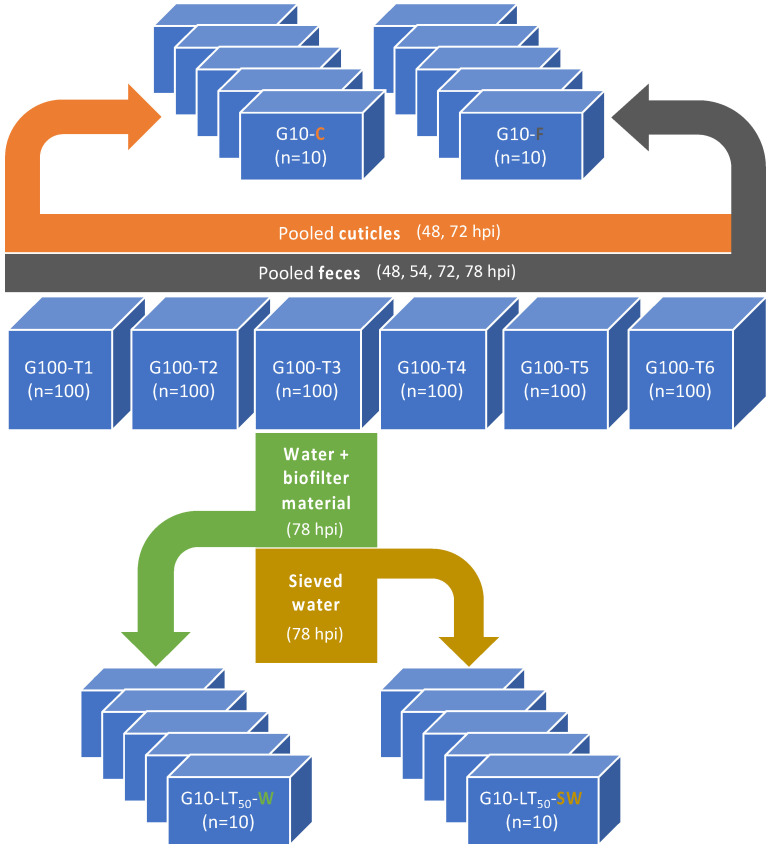
A schematic diagram showing the design of experiment 4: role of molted cuticles, feces, and rearing water in WSSV transmission dynamics: six 290-L WSSV-infected tanks housing 100 shrimp each (G100-T1 to -T6). Molted cuticles were collected from G100-T1 to -T6 at 48 and 72 hpi, pooled, and evenly distributed over the G10-C tanks. Feces were collected from G100-T1 to -T6 at 48, 54, 72, and 78 hpi, pooled, and evenly distributed over the G10-F tanks. Water and biofilter material were collected from G100-T3 (first G100 tank to reach 50% mortality) at 78 hpi and distributed evenly over the G10-LT_50_-W tanks. Then, shrimp were removed from G100-T3, the water was decanted and sieved (250 µm), and then distributed evenly over the G10-LT_50_-SW tanks.

Experiment 4: the experimental design is illustrated in Figure 4. To determine the infectiousness of the different components in the shrimp’s tank environment, six 290-L tanks (G100-T1, -T2, -T3, -T4, -T5, -T6) were each stocked with one hundred PLs with an average weight of 3.5 g to reach a density that corresponds with intensive culture conditions. Shrimp in G100-T1 to -T6 were inoculated with WSSV-positive tissue (stock 2 = 10^8.6^ SID_50_ g^−1^). Mortalities in G100-T1 to -T6 were recorded twice a day and the carcasses were collected for qPCR. Molted cuticles were collected from these tanks at 48 hpi (15 g) and 72 hpi (10 g), pooled after every collection time point, and immediately evenly divided over five replicate 10-L tanks that had been set up, housing ten sentinel shrimp per tank (G10-C(uticles)). Additionally, feces were siphoned and collected from G100-T1 to -T6 at 48, 54, 72, and 78 hpi. Immediately after every collection, these feces were pooled, and a 1 mL sample was stored at −70 °C for qPCR analysis. The rest of the pooled feces sample was divided evenly over another five replicate 10-L tanks, each housing ten sentinel shrimp (G10-F(eces)). Each G10-F tank received 2 g of feces at 48 hpi, 6 g at 54 hpi, 8 g at 72 hpi, and 5 g at 78 hpi. In addition, at 78 hpi, the first out of the six 290-L tanks had surpassed a mortality of 50% (LT_50_). Thus, 50 L of this tank’s water (W) was immediately taken out and evenly divided over five empty 10-L tanks (G10-LT_50_-W). Biofilter material from G100-T3 was also collected and 300 mL was transferred to the biofilter compartment in each replicate G10-LT_50_-W tank. Subsequently, surviving shrimp were removed from G100-T3, euthanized, and the carcasses were stored in −70 °C for qPCR analysis. Then, solid particles in the tank water of G100-T3 were left to sediment for 1 h. Afterwards, 50 L from the upper water layer from which the particles had settled out was suctioned off, following the principle of decantation. This water was sieved (250 µm) (SW) and transferred to the final five 10-L tanks. In these tanks, another group of fifty sentinel shrimp was randomly divided (10 shrimp/tank), making up the fourth and last experimental group G10-LT_50_-SW. Dead and moribund shrimp were collected from these G10 tanks twice a day for qPCR. At the end of the experiment, all surviving shrimp were euthanized. All samples were stored at −70 °C for qPCR analysis. The probability of infection in a population exposed to a certain environmental component was calculated using mathematical Equation (1). Based on the empirical data, the basic reproduction number *R_0_* was calculated (2).

### 2.5. Confirmation of Viral Infection and Shedding

DNA extractions were performed using the DNeasy^®^ Blood & Tissue Kit (Qiagen, Hilden, Germany). The kit’s standard protocol for purification of DNA from tissue was executed to extract DNA from shrimp muscle and gill tissues. For the artificial seawater samples, the standard protocol for DNA extraction from blood was used [49]. Extracted DNA was tested in a quantitative polymerase chain reaction (qPCR) containing 18 µL of a qPCR master mix (PowerUp™ SYBR™ Green Master Mix (Thermo Fisher Scientific, Waltham, MA, USA) in combination with WSSV specific primers targeting the envelope protein gene *vp19* [50]. The qPCR was run in a StepOnePlus™ Real-Time PCR System (Applied Biosystems, Waltham, MA, USA). The obtained amplification and melting curves were analyzed with StepOne™ v2.3 software to validate quantification.

### 2.6. Statistical Analysis

The survival, mortality, and infection data were analyzed statistically using the Log-rank (Mantel–Cox) test. Differences between viral loads were analyzed using a one-way ANOVA, followed by Tukey’s multiple comparisons test. The binomial test was used to evaluate hypotheses about the probability of infection.

## 3. Results

### 3.1. In Vivo Titrations of WSSV-Positive Tissue Inocula

The number of shrimp that died during the *in vivo* titration of the suspensions prepared from the solid inocula can be found in Table S1. WSSV infection was confirmed in a sample of the deceased shrimp by qPCR. Infection was absent in surviving animals. The infectious titers for stock 1 were 10^7.3^ SID_50_ g^−1^, 10^7.7^ SID_50_ g^−1^, and 10^7.7^ SID_50_ g^−1^ ($\bar{x}$ = 10^7.7^ SID_50_ g^−1^), and for stock 2, these were 10^8.8^ SID_50_ g^−1^, 10^8.5^ SID_50_ g^−1^, and 10^8.5^ SID_50_ g^−1^ ($\bar{x}$ = 10^8.6^ SID_50_ g^−1^).

### 3.2. In Vivo WSSV Challenges

#### 3.2.1. Characterization of WSD Progression

**Figure 5:**
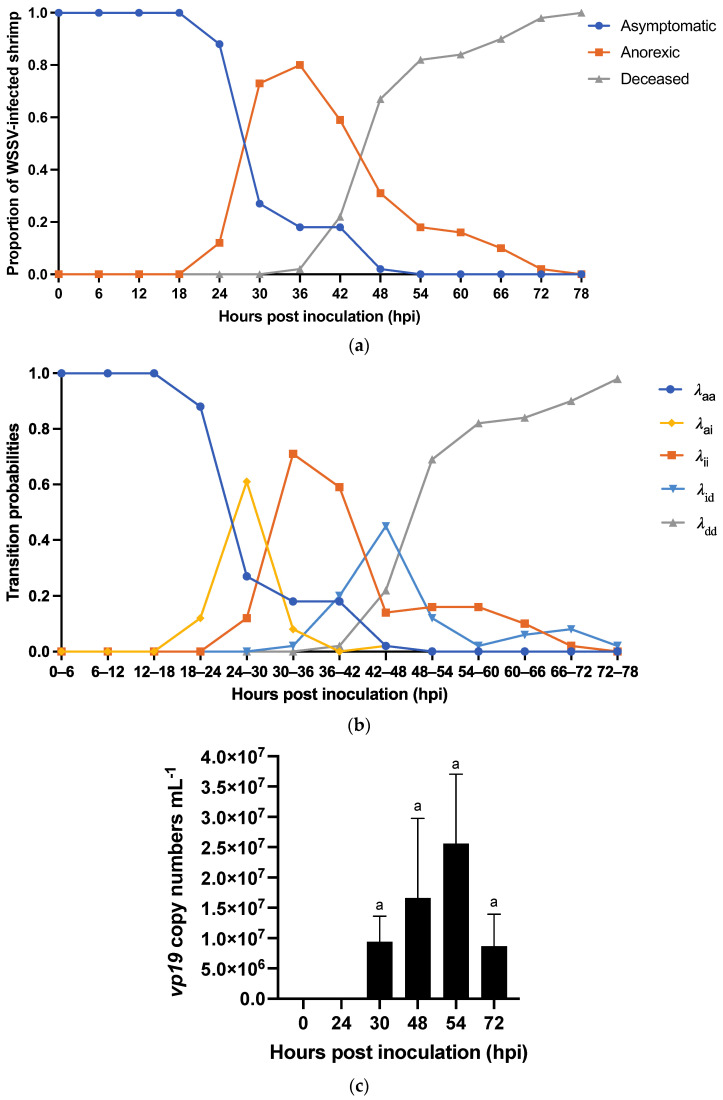
(**a**) Characterization of WSD progression in WSSV-infected shrimp: proportion of WSSV-infected shrimp in a particular state of disease at a specific time point post inoculation with WSSV-positive tissue inoculum. The three states of disease were: (1) asymptomatic, (2) ill (anorexic), (3) deceased. (**b**) Transition rates or probabilities per time-interval post-inoculation. *λ*_aa_: probability that an asymptomatic individual will remain asymptomatic; *λ*_ai_: probability that an asymptomatic individual will transverse into a state of illness (anorexia); *λ*_ii_: probability that an anorexic individual will remain anorexic; *λ*_id_: probability that an individual will die after being anorexic; *λ*_dd_: probability that an individual is deceased. (**c**) Analysis of the WSSV DNA shedding of WSSV-infected shrimp: concentration of *vp19* genetic material in water samples taken at 0, 24, 30, 48, 54, and 72 hpi from a tank housing a *L. vannamei* shrimp suffering from WSD. This individual developed anorexia at 24 hpi and died at 54 hpi. It was removed from the tank shortly after its death. The letter ‘a’ indicates that the differences between time points were not significant (*p*-value > 0.05). Similar results were obtained for the other tanks (Table S2).

Experiment 1a: WSSV infection was confirmed by qPCR in a sample of deceased shrimp, while WSSV was absent in sampled survivors and negative controls. Overall, the probability of infection after inoculation was 0.34 ± 0.14. In the shrimp that were infected, the WSSV Thai-1 strain was shown to have an incubation period between 24 and 54 hpi, since the earliest cases of anorexia were observed at 24 hpi, and the last asymptomatic shrimp had become anorexic at 54 hpi (Figure 5a). Mortality first occurred at 36 hpi and ceased at 78 hpi when final cumulative mortality reached 34%. During this experiment, all WSSV-infected shrimp progressed through three states of disease: an asymptomatic state, a state of illness where they presented with clinical symptoms (i.e., anorexia), and finally death. Transitions through the states were irreversible. However, individuals could remain in a particular state over time (Figure 5b). The transition rates (*λ*) show that the risk for an infected shrimp to move from the asymptomatic state into a state of anorexia was the highest between 24 and 30 hpi (0.61). Death after a period of anorexia was the most probable occurrence between 42 and 48 hpi (0.45). Finally, at 72–78 hpi, the probability of being in the final state was 1.0 (Figure 5b).

Experiment 1b: in this experiment, it was demonstrated that WSSV-infected shrimp were shedding viral DNA over the course of the disease, and this shedding reached a peak around the time of death. Eleven shrimp out of forty (27.5%) became infected, while the others (72.5%) remained clinically healthy. Nine out of these eleven shrimp (81.8%) showed anorexia at 24 hpi, while the other two (18.2%) showed anorexia at 48 hpi. In all nine tanks housing anorexic shrimp at 24 hpi, WSSV DNA could be detected within 6 h after the appearance of symptoms. In one of the tanks, housing anorexic shrimp at 48 hpi, WSSV DNA was detected 24 h before the onset of anorexia, while in the other tank it was detected when the anorexic shrimp had died. Though the differences between the sampling time points were not significant when WSSV DNA in the tank water was quantified, the following trend (recurring pattern) was observed: an increase of the DNA concentration from the time of the first DNA detection to the death (and removal) of the infected shrimp. This was followed by a decline of the DNA concentration at the time point measured right after death. This dynamic is illustrated in Figure 5c.

#### 3.2.2. WSSV Infection in Relation to Population Density

Experiment 2: the threshold density for the definite occurrence of a WSD epidemic in our infection model was found to be 10 shrimp per 10 L. By testing different stocking densities, we demonstrated that the initial probability or risk of infection in a tank following the group inoculation varied between the different stocking densities (Table 1). However, once WSD manifested clinically in a tank, all shrimp in that tank were invariably infected over a period of 186 hpi, and all infections were lethal. In other words, the final survival after inoculation in any given tank was either 100% or 0%. WSSV infection occurred significantly more frequently (*p*-value < 0.05) in tanks with higher stocking densities. The infection risk was 1.0 in the tanks that housed 10 shrimp (G10). Shrimp housed in groups of five, three, and two, as well as individually housed shrimp, were at a significantly lower risk (*p*-value < 0.05) i.e., 0.60, 0.59, 0.36, and 0.18, respectively (Table 1).

#### 3.2.3. The Effect of Isolation on WSSV Epidemic Dynamics

**Figure 6:**
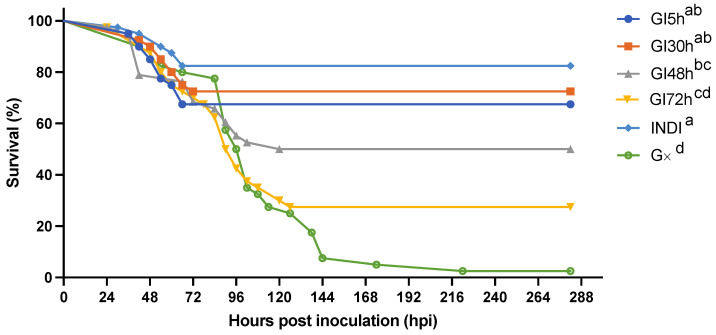
Effect of isolation on the survival curves of groups of *Litopenaeus vannamei* challenged with WSSV-infected tissue inoculum: values of INDI consist of 40 tanks housing one shrimp each. Values of groups GI5h, GI30h, GI48h, GI72h and G∞ represent the average of 4 replicates consisting of 10 shrimp each. Different letters indicate significant differences (*p*-value < 0.05) between the groups.

Experiment 3: there was a positive correlation between the final mortality in the experimental groups GI5h, GI30h, GI48h, and GI72h and the duration of the time that they were housed together before being isolated. In other words, the longer shrimp were housed in groups, the higher the mortality. The survival curves of the experimental groups GI5h, GI30h, GI48h, GI72h, INDI, and G∞ are displayed in Figure 6 and a summary of the *p*-values can be found in Table 2. The highest survival occurred in INDI (82.5%), while only 2.5% of shrimp survived in G∞. Survival in INDI (82.5%), GI5h (67.5%), and GI30h (72.5%) was significantly higher than in GI72h (27.5%) and G∞ (2.5%). Survival was also significantly higher in INDI (82.5%) compared to GI48h (48.8%) (*p*-value < 0.05). Group GI48h (48.8%) in turn ended up with a significantly higher survival than G∞ (2.5%) (*p*-value < 0.05), but there was no statistically significant difference between survival of GI48h (48.8%) and GI72h (27.5%) (*p*-value > 0.05). The onset of mortality occurred at 24 hpi for GI48h and GI72h. For INDI, mortality started at 30 hpi, while the first mortality for GI5h was recorded at 36 hpi. Finally, at 42 hpi, mortality was also first observed in GI30h and G∞. The median lethal time (LT_50_) was reached in GI48h at 120 hpi, in GI72h at 90 hpi, and in G∞ at 96 hpi. Mortality ceased the earliest in GI5h and INDI at 66 hpi, closely followed by a cessation in GI30h at 72 hpi. The last mortalities of GI48h and GI72h occurred at 120 and 126 hpi, respectively, while the final mortality in G∞ was recorded at 222 hpi (Figure 6). WSSV infection was confirmed by qPCR in a sample of deceased shrimp, while WSSV was absent in sampled survivors and negative controls.

**Figure 7:**
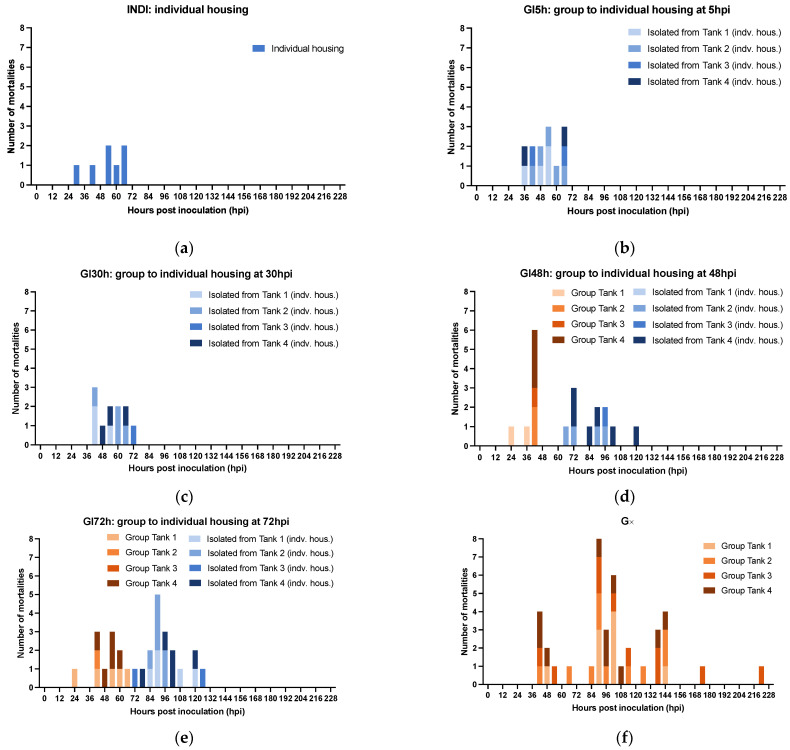
Effect of isolation on epidemic curves showing mortality in groups of *Litopenaeus vannamei* challenged with WSSV-infected tissue. INDI consisted of 40 individually housed shrimp. GI5h, GI30h, GI48h, GI72h, and G∞ consisted of 4 replicate groups of 10 shrimp each. (**a**) INDI was housed individually throughout the whole experiment; (**b**–**e**) Groups GI5h, GI30h, GI48h, and GI72h were isolated at different time points post inoculation (5, 30, 48, and 72 hpi, respectively). (**f**) G∞ remained housed in group throughout the whole experiment.

An epidemic curve is a histogram that displays the number of disease cases in animals during an outbreak by times of onset of illness. Here, the survival/mortality was used as an indicator for WSD because our study of the natural course of WSD demonstrated that all diseased animals invariably transition into the deceased state (Figure 5a,b). The epidemic curves showing mortality in the different experimental groups are displayed in Figure 7. The results of this experiment showed that the disease in our infection model was not propagated in the inoculated populations up to 30 hpi. However, there was disease propagation in groups that remained in group housing until 48 hpi or later. INDI, GI5h, and GI30h follow an epidemic curve pattern of a point source outbreak, while GI48h, GI72h, and G∞ present a propagated outbreak pattern. In Section 3.2.1, we established that the usual incubation period for WSSV Thai-1 is 24–54 hpi, and that the probability of death after individual infection with WSSV Thai-1 was 1.0 at 78 hpi. In the current challenged groups, all shrimp were exposed to WSSV-infected tissue at the same point in time. However, all deaths in groups INDI, GI5h, and GI30h occurred within 72 hpi, while the duration of the outbreaks in GI48h, GI72h, and G∞ were extended, ending at 120, 126, and 222 hpi, respectively. The curve of GI48 h has one clear upward slope at 42 hpi followed by a gradual downward slope. However, this slope does not cover the 78h-period. The epidemic curve for GI72h peaks twice, once around 42–54 hpi and a second time at 90 hpi. The second peak occurs after 78 hpi and is larger than the first one. The epidemic curve of G∞ shows three larger peaks at 42, 90, and 144 hpi and two smaller peaks at 174 and 222 hpi. The distance between these peaks gives a rough estimate of the incubation period. For INDI, GI5h, and GI30h, the estimated *R*_0_ was 0. The average estimated *R*_0_ of the four replicate tanks from GI48h was 0.6 ± 0.4, for GI72h it was 2.2 ± 2.2, and finally for G∞, the *R*_0_ was 3.4 ± 2.1.

**Figure 8:**
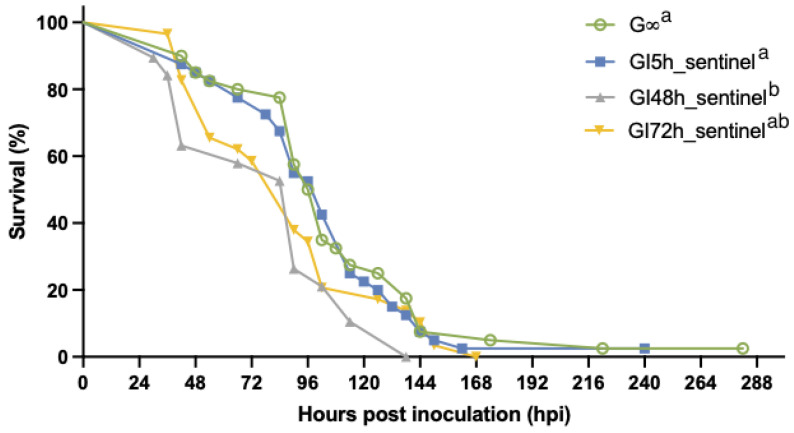
Survival curves of groups of *Litopenaeus vannamei* sentinels that repopulated the vacated group tanks of GI5h, GI48h, and GI72h in which a WSSV outbreak had occurred. The survival curve of G∞, a group of *Litopenaeus vannamei* challenged with WSSV-infected tissue inoculum was included in this graph. Zero hpi is the time of entrance into the tanks. Values of groups GI5h_sentinel, GI48h_sentinel, and GI72h_sentinel and G∞ represent the average of 4, 2 and 3 infected tanks housing 10 shrimp each. Value of G∞ represents the average of 4 replicate tanks housing 10 shrimp each. Different letters indicate significant differences (*p*-value < 0.05).

When sentinels were used to repopulate tanks after the removal of the originally inoculated shrimp that had remained in these tanks in group housing for 30, 48 and 78 hpi, our results show that the initial probability of infection in the sentinel populations increased between 30 and 72 hpi. However, the probability of infection (=1.0) in the GI5h_sentinel populations was equal to the probability of infection in the G∞ populations. It should be noted that both GI5h_sentinel and G∞ were housed in water in which shrimp had been feeding on WSSV-infected tissues, though the first group had not ingested these tissues while the latter group had. The probability of WSSV infection (=1.0) in GI5h_sentinel was significantly higher (*p*-value < 0.05) than for the GI30h_sentinel, GI48h_sentinel, and GI72h_sentinel groups (Table 3). None of the shrimp in the GI30h_sentinel populations were infected after transfer to the GI30h group tanks. Two out of four GI48h_sentinel populations and three out of four GI72h_sentinel populations experienced a WSSV outbreak in their tanks (Table 3). Once WSD manifested in any given tank, the final survival was close to 0% (Figure 8). Survival was 100% in the tanks that were not infected.

The survival curves of the sentinel tanks that were affected by WSD are presented in Figure 8 for each experimental group. The survival curve of the two WSSV-infected sentinel tanks of GI48h_sentinel was significantly different (*p*-value < 0.05) from the curves of the four infected GI5h_sentinel tanks, the three infected GI72h_sentinel tanks, and the four infected tanks of the orally inoculated group G∞ (Figure 8). The onset of mortality in GI5h_sentinel was at 42 hpi. For GI48h_sentinel, the first mortalities occurred at 30 hpi, and for GI72hpi at 36 hpi. Time point 102 hpi marked the LT_50_ for GI5h_sentinel, while the LT_50_ was reached at 90 hpi for GI48h_sentinel and GI72h_sentinel. The final case of mortality in GI5h_sentinel was recorded at 162 hpi. Mortality ceased in GI48h_sentinel at 138 hpi and at GI72h_sentinel at 168 hpi. WSSV infection was confirmed by qPCR in a sample of deceased shrimp, while WSSV was absent in sampled survivors and negative controls.

**Figure 9:**
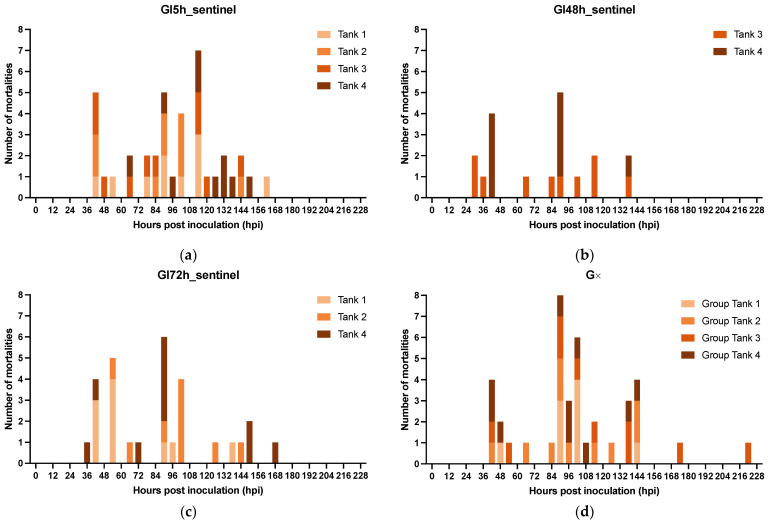
Epidemic curves showing mortality in groups of *Litopenaeus vannamei* sentinels that repopulated the vacated group tanks of GI5h, GI48h, GI72h and that suffered a WSSV outbreak. GI5h, GI48h, GI72h and G∞ consist of 4, 2, 3, and 4 infected tanks housing 10 shrimp each: (**a**) GI5h_sentinel, (**b**) GI48h_sentinel, and (**c**) GI72h_sentinel. (**d**) The epidemic curve of G∞, a group of *Litopenaeus vannamei* challenged with equal doses of WSSV-infected tissue inoculum was included in this Figure. 0 hpi is the time of entrance into the tanks.

If WSD manifested itself in one of the sentinel tanks, the resulting epidemic pattern and disease propagation in these infected tanks of GI5h_sentinel, GI48h_sentinel, and GI72h_sentinel was comparable to the epidemic curve of the WSSV-tissue inoculated tanks in G∞ (Figure 9). Every curve demonstrates successively larger peaks, with the first one occurring around 42 hpi, and the second and largest one appearing between 90–114 hpi. This is followed by a gradual downward slope. Based on the empirical data, the average estimated *R*_0_ of the four GI5h_sentinel tanks was 2.5 ± 1.1. The two GI48h_sentinel tanks had an average estimated *R*_0_ of 1.3 ± 0.3 and for GI72h_sentinel, it was 2.0 ± 1.3.

#### 3.2.4. Role of Molted Cuticles, Feces, and Rearing Water in WSSV Transmission Dynamics

**Figure 10:**
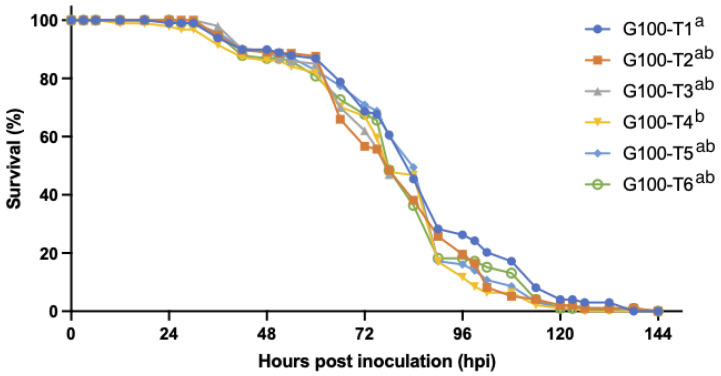
Survival curve of *Litopenaeus vannamei* challenged with WSSV-infected tissue in six 290-L tanks. Shrimp were housed in groups of a hundred per tank. The curve of G100-T3 was discontinued at 78 h when mortality exceeded 50%. Different letters indicate significant differences (*p*-value < 0.05) between tanks.

Experiment 4: the survival in G100-T1, T2, T4, T5, and T6 reached 0% at 144 hpi (Figure 10). Only the survival curve of G100-T4 was significantly steeper than the curve of G100-T1 (*p*-value = 0.041). The survival in G100-T3 was 47% at 78 hpi (after which the remaining shrimp were euthanized) and between 0hpi and 78 hpi the survival curve was not significantly different from the survival curves from any of the other tanks (*p*-value > 0.05). Mortality set in at 24 hpi in G100-T1 and T4, at 27 hpi in T5 and T6, and at 36 hpi in T2 and T3. The median lethal time was reached first in T2, T3, T4, and T6 at 78 hpi. At 84 hpi, T1 and T5 also reached their median lethal time. Mortality ceased at 126 hpi in T4, at 138 hpi in T1 and T5, and at 144 hpi in T2 and T6. WSSV infection was confirmed by qPCR in a sample of deceased shrimp, while WSSV was absent in sampled survivors and negative controls.

**Figure 11:**
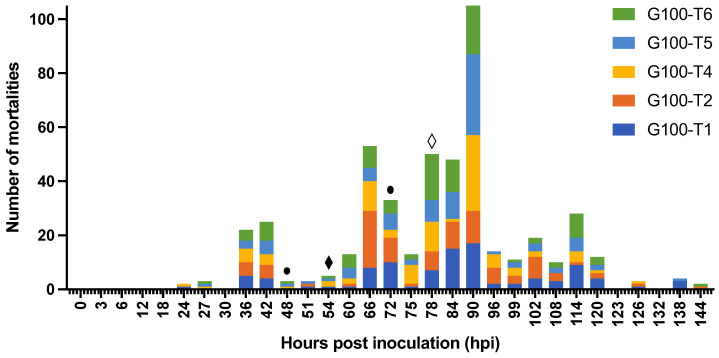
Epidemic curve showing mortality in five WSSV challenged 290-L tanks housing one hundred *Litopenaeus vannamei* shrimp each; ● collection of molted cuticles and feces; ♦ collection of feces; ◊ collection of feces, water and biofilter material, decanted and sieved water.

The epidemic curve of the G100-T1 to T6 tanks has a propagated outbreak pattern (Figure 11). The peaks in this curve become progressively taller, each being approximately one incubation period apart. The onset of the epidemic occurs at 24 hpi, and the first peak appears at 36–42 hpi. The graph assumes a third peak at 66 hpi, 42 h after the onset of the epidemic, and the fourth and largest peak arises at 90 hpi, 48 h after the first peak at 36–42 hpi. The subsequent peaks start to diminish over time. The average *R*_0_ of 2.0 ± 0.4 of the five tanks was estimated based on the empirical data.

Exposure to WSSV-contaminated rearing water collected from infected tanks resulted in a significantly higher probability of infection in sentinel populations than exposure to feces or molted cuticles from the same infected tanks. The probability of infection in relation to exposure to potential WSSV-contaminated environmental components is displayed in Table 4. None of the G10-C were infected. One tank out of five was infected in G10-F (0.2). In comparison, the tanks of G10-LT_50_-W had a significantly higher infection risk of 1.0 (*p*-value < 0.05), and, in the G10-LT_50_-SW tanks, the risk of infection was also significantly higher at 0.8 (*p*-value < 0.05) (Table 4).

**Figure 12:**
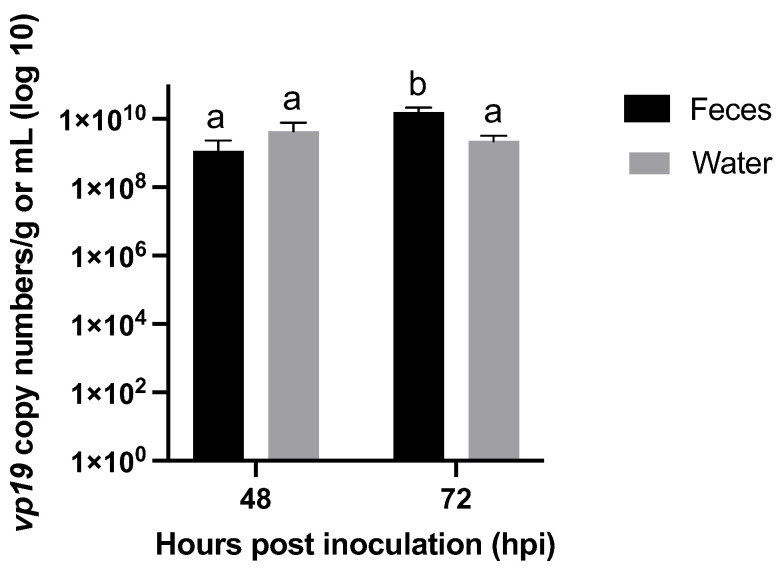
Average concentration of *vp19* copy numbers in pooled feces and water samples taken from six 290-L tanks housing *Litopenaeus vannamei*, two and three days after these animals were challenged with WSSV-infected tissue inoculum. Different letters indicate significant differences (*p*-value < 0.05) between components and time points.

When the concentration of *vp19* copy numbers in the pooled feces was quantified, there was a significant increase (*p*-value < 0.05) between the feces samples taken on the second day (1.2 × 10^9^ ± 8.52 × 10^8^ copy numbers/g) and the third day (1.6 × 10^10^ ± 4.36 × 10^9^ copy numbers/g) after inoculation (Figure 12). Moreover, the *vp19* copy numbers in the feces on the third day after inoculation were significantly higher than in the water sampled on the second (4.3 × 10^9^ ± 3.22 × 10^8^ copy numbers/mL) and the third day (2.2 × 10^9^ ± 8.77 × 10^8^ copy numbers/mL) post inoculation. There was no significant difference between the average *vp19* copy numbers in the water collected two and three days after inoculation (*p*-value > 0.05).

## 4. Discussion

In this study, the dynamics of WSSV epidemics and transmission were elucidated in controlled experimental infection models. An oral inoculation method that was established for the WSSV Thai-1 strain by Thuong et al. [22] was used to emulate natural WSSV infection in the field [13,14]. The WSSV Thai-1 strain has been extensively examined in *L. vannamei* [22,36,41,43,44,51]. It was found to be highly virulent [36]. However, a study to characterize the natural course of WSD caused by this strain via natural feeding on WSSV-infected tissues was never conducted. Hence, the first experiment of this study established the rapid time course of this disease with an onset of clinical symptoms at 24–30 hpi, and the probability of death already being at its highest between 42–48 hpi. Moreover, the disease irreversibly leads to death no later than 78 hpi. These findings were imperative to the accurate analysis of the epidemic patterns of spread in our third experiment. Furthermore, these observations imply that a time-based intervention in the event of an outbreak caused by a virulent WSSV, such as WSSV Thai-1, would be the most appropriate, and this must be considered when developing countermeasures [52].

During the first experiment, most WSSV-infected animals started shedding viral DNA in the water within 6 h of disease onset. Additionally, our data indicated that viral DNA shedding intensified over the course of the disease, so that the WSSV DNA concentration in the tank water reached its peak around the time of death. This suggests that there was a correlation between WSD severity and the viral DNA shedding rate of infected shrimp, which is supported by another study that was recently published [53]. It should be noted, however, that using PCR methods does not give information about the detected virus’s ability to infect a susceptible host [24]. Thus, direct conclusions between WSD severity and the shedding of infectious virions cannot be made. Previous studies have implied that if infected animals would shed infectious virions, these may remain viable and infective for 12–19 days [24], while WSSV DNA may even persist for over 20 months [54]. This might indicate that a WSSV-infected shrimp could, in theory, be a risk factor for other susceptible conspecifics between 24 hpi and 19 days beyond its death. However, in our experiment, a decline in DNA concentration was detected after the shrimp’s removal from the tank, suggesting that the infectious period was possibly much shorter. This should be considered when analyzing the pattern of a WSSV epidemic.

High densities have been identified as an important risk factor for WSD [55]. To develop a reproducible experimental group infection model that would be suited to use for epidemiological studies, we aimed to find a threshold density at which a WSD epidemic would always occur. Indeed, when increasing the experimental population density from 2 individuals per 10 L to 10 individuals per 10 L in our second experiment, the risk of initial infection in a tank after exposure to WSSV-infected tissues increased significantly. However, once WSD manifested in at least one shrimp in a tank, all shrimp in that 10-L tank were invariably lethally infected during the experiment, regardless of the stocking density. Thus, in this setup, the level of secondary host-to-host WSD transmission after the initial inoculation could not be related to the stocking density. In the field, it is generally accepted that the risk of transmission increases with stocking density, possibly due to the greater number of contacts between shrimp spreading the pathogen [55,56,57]. However, not all studies have succeeded in associating a greater prevalence of WSD with high stocking densities [57,58,59]. Nevertheless, a reproducible experimental group infection model with a stocking density of 10 shrimp per 10 L that could be used for our epidemiological experiments was established.

Isolation has been used to elucidate transmission dynamics of well-known infectious diseases, including COVID-19, avian influenza, and Ebola virus disease [60,61,62]. In turn, the effect of isolation on WSSV epidemic and transmission dynamics was studied in our third experiment. First, it was shown that the final survival in the experimental populations that were exposed to WSSV-infected tissue and then isolated up to 30 hpi was not significantly lower than the survival of the shrimp that had been challenged individually. This shows that shrimp behaviors associated with cohabitation did not significantly affect the number of shrimp that became infected during the first 30 h of the challenge in group. Moreover, the epidemic curves of these three groups show that none of the shrimp from these populations died later than 72 hpi. This strongly suggests that all lethal infections in these groups were caused because of the initial inoculation and not by a secondary host-to-host transmission [63]. Indeed, even though our study of the disease course in the individual infection model showed that by 30 hpi, most infected shrimp were symptomatic and shedding viral DNA, the viral transmission was apparently not occurring yet in the group infection model by that time point. When inoculated shrimp populations remained in group housing for more than 48 h, we observed an increasingly significant negative impact on the groups’ final survival the longer they stayed together. The epidemic curves of these populations did not cover one 78 h-period as with the epidemic curves of groups that were separated earlier. They displayed one or multiple distinct peaks, followed by a downward slope, implying that in these groups the disease was propagated before the animals were isolated in individual tanks [63].

First, these results show that the isolation of the animals acted as an adequate control measure to reduce the size of the WSD epidemic in this experimental setting. The earlier this measure was taken, the more effective it was to decrease the number of secondary cases and to prevent a fully developed outbreak. Indeed, this indicates again that a time-based intervention in case of an outbreak in the field, for instance by administration of effective prophylactic treatments, might still prevent mass mortalities [52].

Second, these findings demonstrate that the first cases of host-to-host transmission start occurring between 30 and 48 h after inoculation. This was happening in parallel with the occurrence of the first mortalities. This observation appears to support the work of Soto and Lotz [17] because it shows that co-habitation with live sick shrimp for 30 h in our setup did not result in WSSV transmission. In other words, our results suggest that host-to-host transmission is only occurring around the time of death of an infected individual.

**Figure 13:**
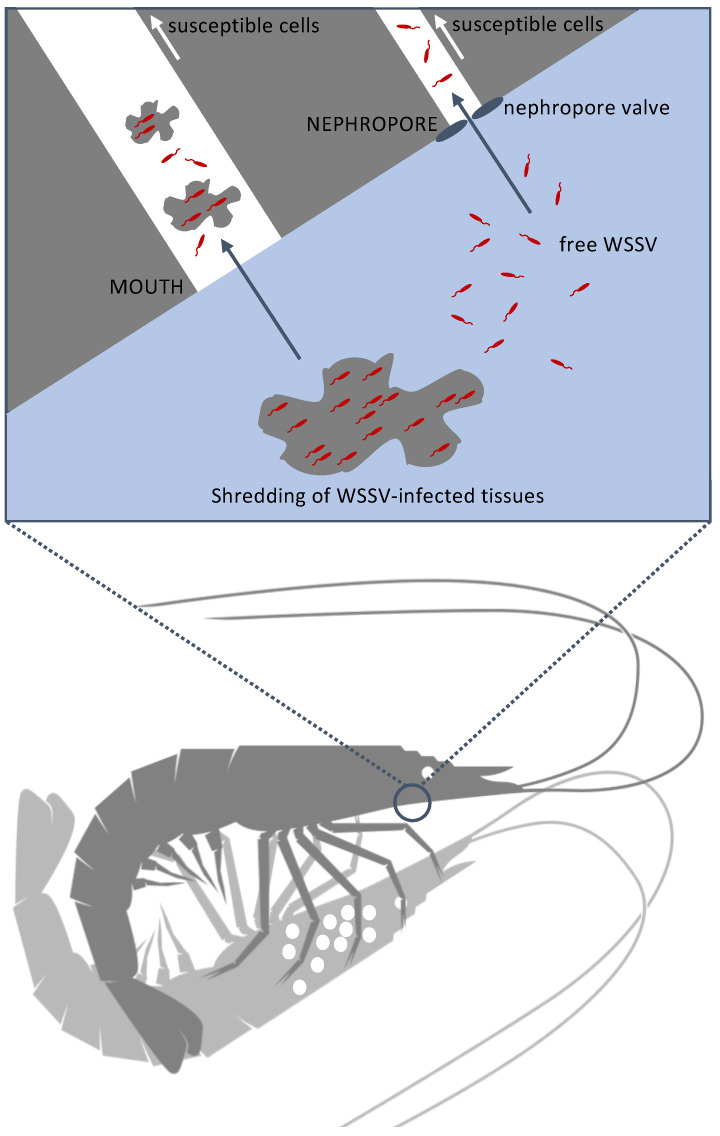
Hypothetical model for water-borne WSSV transmission in shrimp.

Additionally, in the third experiment, it was confirmed that sentinel populations could be infected by housing them in the vacant tanks that had been previously occupied by an infected population. Thus, it confirmed that these tanks contained infectious WSSV at the time of the sentinels’ entrance and that the indirect environmental transmission route is relatively effective [15]. The data showed that the longer the infected population remained in the tanks before they were removed (between 30 and 72 hpi), the higher the infection risk (0.0 to 0.75) became for the group of sentinels that repopulated those tanks. This indicated the presence of an increasing concentration of infectious viral particles over time. However, the infection risk was equal (1.0) for the sentinel populations that repopulated the vacated tanks at 5 hpi and the tissue-inoculated shrimp populations that remained in group housing throughout the whole experiment. Moreover, the average survival curves of both groups were not significantly different from each other. This was an unexpected observation because (1) the results of the natural history study had shown that infected shrimp were not shedding detectible quantities of viral DNA yet at 5 hpi, and (2) WSSV-infected tissues from the inoculation of the first population were confirmed to be completely consumed before the sentinels entered the tanks at 5 hpi. Still, the results showed that infectious WSSV was present in these tanks, which raised the question of where this infectious virus originated from. What could be confirmed is that both the sentinel populations that entered the vacated tanks at 5 hpi (a) and the infected tissue-inoculated populations that were not isolated (b) found themselves in water in which shrimp had been feeding on WSSV-positive tissues before 5 hpi. However, one group (b) had ingested these WSSV-infected tissues themselves while the other group (a) had not. In other words, ingestion of WSSV-infected tissues did not cause a significantly higher number of index cases in the experimental setup of this study. This result could imply that WSSV spread through cannibalism, defined as the consumption of an infected individual, was far less important. Indeed, this theory is supported by the research of De Gryse et al. [43], which proved that the *per os* route as a potential entry point for WSSV is very inefficient, especially when compared to the nephrocomplex route. When cannibalism as a transmission mode for WSSV is discussed, it is worth mentioning that diseases transmitted predominantly by cannibalism are rare because the epidemiological conditions necessary for its spread, especially group cannibalism, are rarely met in natural populations [64]. In the animal kingdom, cannibalism is generally a one-on-one interaction in which a larger and stronger individual kills and consumes a smaller and weaker conspecific [65]. Under these conditions, cannibalism is likely to be an ineffective mode of disease transmission because, in one-on-one cannibalism, the R_0_ of a cannibalistically transmitted disease must be less than unity. For disease spread through cannibalism, group cannibalism by multiple individuals on one victim is a necessary (albeit not always sufficient) precondition for disease. In truth, cannibalism was implicated as the major transmission mode for only two pathogens: prion transmission in humans (Kuru) [66] and transmission of the protozoan *Sarcocystis* in lizards [64,67]. In all other cases, even for shrimp diseases [68,69,70,71,72,73,74,75,76], there were alternative transmission modes reported [64]. It should be noted though that both diseases that primarily transmit through cannibalism occur in terrestrial species. Shrimp, on the other hand, are aquatic. When they partake in cannibalism one-on-one or even potentially in group, it takes place in water, which is a different kind of medium. WSSV transmission by cannibalism of infected cadavers has traditionally been equated to peroral transmission [17]. However, in a medium such as water, this could but might not necessarily be the case. Shrimp have been described to eat very wastefully [77]. The results of our experiment strongly suggested that the act of cannibalism in water could potentially disseminate infectious WSSV particles, immersing the cannibal and other shrimp in its vicinity, and facilitating virus transmission. Additionally, in this scenario, it should be remarked that even though shrimp in the vicinity of a cannibalizing shrimp could become infected, this should not mean that the occurrence of infection in the cannibal is certain. This could potentially be an explanation for the contradicting results from Soto and Lotz [17] and Tuyen et al. [15], who used different setups for their experiments. The authors of the former study carried out a co-habitation experiment (co-habitation with sick shrimp without cannibalism) and an ingestion (cannibalism) experiment in separate aquariums, concluding that transmission by ingestion was seemingly the most important mode of transmission [17]. This conclusion suffered from the unconscious bias that because cannibalism involves the ingestion of infectious tissues, it is this ingestion that causes the transmission of WSSV. When Tuyen et al. [15] conducted their pairwise co-habitation experiment, all shrimp were housed in the same tank sharing the same rearing water. The authors concluded that indirect water-borne contact was more important for the transmission of WSSV than direct contact. However, they also considered cannibalism to only be a co-factor of direct contact transmission, and they did not associate it with indirect environmental transmission. Based on the results of our third experiment, we uniquely postulate that the act of cannibalism could facilitate indirect environmental transmission because the act of chewing potentially releases multiple virus particles into the water, which thereby determines the virus load. Meanwhile, ingestion of infected material by itself appears to present a less important component of transmission than previously assumed. The entry process of WSSV into the shrimp at the cellular and molecular levels is not understood, but we hypothesize that water-borne WSSV-virions might enter via the nephropores or *per os* to infect susceptible cells (Figure 13) [43,78]. However, further research is needed to pinpoint the underlying mechanisms.

In the fourth experiment of this study, the possible role of molts, feces, and rearing water in the transmission of WSSV was investigated. These materials were collected from infected 290-L tanks housing one hundred shrimp each that were inoculated with the second WSSV stock, which had a higher infectious titer than the first stock. This could explain the rapid spread of the disease in these infected tanks. It was demonstrated that infected rearing water collected from these tanks resulted in the highest risk of infection in sentinel populations and that WSSV transmission was water-borne. Exposing sentinels to decanted and sieved water resulted in a slightly lower risk of infection compared to exposure to water and biofilter material. An explanation for this observation might be offered by the findings from several other studies that discovered WSSV could be detected in zooplankton [23] and temporarily attach to phytoplankton when the virus concentration in the water was high [11,79,80]. If plankton acted as a vector for WSSV in our experimental setup, decanting and sieving rearing water and foregoing the addition of biofilter material might have reduced this vector concentration and therefore possibly also the risk of infection. Exposing sentinels to feces produced by shrimp from infected populations, did not appear to substantially facilitate WSSV transmission, like the rearing water from the infected populations did. Even though the viral DNA load in these feces was relatively high and at one point even significantly more concentrated than the viral DNA load in the tank water, our results imply that WSSV-infected animals were not substantially shedding infectious virus in their feces. The presence of this DNA might not have been due to viral shedding though. Instead, it could potentially have resulted from non-anorexic shrimp cannibalizing their moribund or deceased WSSV-infected conspecifics. Gastrointestinal passage of this infected meal might have caused a loss of viral infectivity [81]. The major target tissues of WSSV are of ectodermal and mesodermal embryonic origin, especially the cuticular epithelium and subcuticular connective tissues [82]. However, exposing sentinels to molted cuticles collected from infected populations did not result in infection. It should be noted that the molted cuticles as well as the pooled feces originated from infected populations that were ten times the size of the sentinel populations exposed to these materials. Therefore, we assumed that the quantity of these materials (cuticles, feces) would also exceed the normal quantity that sentinel populations of that size would produce themselves during a WSD outbreak. The fact that those materials in these relatively large quantities did not convincingly result in WSSV infection, implies that their role in WSSV transmission is potentially rather limited. It should also be considered that possible saturation of the feces by the infected tank water could not be prevented in this experimental setup. We could not rule out that this might have caused the WSD outbreak in one of the five tanks that were exposed to these feces.

Future research should focus on elucidating the transmission dynamics of WSD on a cellular and molecular level by investigating the potential sites of infection, WSSV replication, and shedding. Examining differences in the genotypes of WSD susceptible and WSD resistant lines of *L. vannamei* might also lead to discoveries on the molecular mechanisms that play a role during WSSV transmission.

## 5. Conclusions

The WSSV Thai-1 strain had an incubation period of 24–54 hpi, and an irreversible disease progression leading to death within 78 hpi. Shrimp infected with this strain were shedding viral DNA over the course of the disease, and this shedding reached a peak within 12 h of the time of death. The threshold density for the occurrence of a WSD epidemic in a group infection model was 10 shrimp per 10 L. At this density, the first cases of host-to-host transmission occurred between 30 and 48 hpi in parallel with the occurrence of the first mortalities. Ingestion of WSSV-infected tissues did not significantly increase the number of index cases during an epidemic compared to immersion into water in which cannibalism had occurred. This could indicate that direct WSSV transmission, through the ingestion of infected tissues, plays a less important role in WSSV transmission than previously thought. Moreover, the investigation of the role of other environmental components (water, feces, molts), showed that exposing sentinels to rearing water collected from WSSV-infected tanks resulted in a significantly higher probability of infection than exposure to feces or molts. Therefore, we postulate that the occurrence of cannibalism of infected shrimp contributes to indirect water-borne WSSV transmission by the spread of free infectious viral particles.

## Figures and Tables

**Table 1:** Probability or risk of WSSV infection in relation to the population density. Different letters indicate significant differences between groups (*p*-value < 0.05).

Group (*n* = 50)	Number of Shrimp/10 L	Number of Replicates	Probability of Infection
G10	10	5	1.00 ^a^
G5	5	10	0.60 ^b^
G3	3	17	0.59 ^b^
G2	2	25	0.36 ^c^
INDI	1	40	0.18 ^d^

**Table 2:** Summary of *p*-values from pairwise comparisons of the survival curves of groups GI5h, GI30h, GI48h, GI72h, INDI and G∞.

Group	GI5h	GI48h	G∞	INDI	GI72h
GI48h	0.195 ^ns^	-	-	-	-
G∞	<0.001 *	0.001 *	-	-	-
INDI	0.116 ^ns^	0.004 *	<0.001 *	-	-
GI72h	0.004 *	0.111 ^ns^	0.204 ^ns^	<0.001 *	-
GI30h	0.578 ^ns^	0.064 ^ns^	<0.001 *	0.293 ^ns^	<0.001 *

^ns^ non-significant. * *p*-value < 0.05.

**Table 3:** Probability or risk of WSSV infection in groups of *Litopenaeus vannamei* sentinels that repopulated the vacated group tanks of GI5h, GI30h, GI48h, and GI72h. Different letters indicate significant differences (*p*-value < 0.05) between groups.

Group	№ of Shrimp 10 L^−1^	№ of Replicates	№ of Infected Populations/ Total № of Populations	Probability of Infection
GI5h_sentinel	10	4	4/4	1.00 ^a^
GI30h_sentinel	10	4	0/4	0.00 ^b^
GI48h_sentinel	10	4	2/4	0.50 ^c^
GI72h_sentinel	10	4	3/4	0.75 ^c^

**Table 4:** Probability or risk of WSSV infection in sentinels in relation to exposure to potentially WSSV-contaminated environmental components. Different letters indicate significant differences (*p*-value < 0.05) between groups.

Group (*n* = 50)	№ of Shrimp 10 L^−1^	№ of Replicates	Environmental Component	№ of Infected Populations/ Total № of Populations	Probability of Infection
G10-C	10	5	Molted cuticles	0/5	0.00 ^a^
G10-F	10	5	Feces	1/5	0.20 ^b^
G10-LT_50_-W	10	5	Water and biofilter material	5/5	1.00 ^c^
G10-LT_50_-SW	10	5	Water (no particles > 250 µm)	4/5	0.80 ^c^

## Data Availability

Data is contained within the article, represented graphically.

## Supplementary Materials

The following supporting information can be downloaded at: https://www.mdpi.com/article/10.3390/v15091824/s1, Table S1: Infectious titers (SID_50_ g^–1^) of two solid inoculum stocks through i.m. injection of *Litopenaeus vannamei* postlarvae. Table S2: Concentration of *vp19* in water samples taken at 0, 24, 30, 48, 54, and 72 hpi from tanks housing a *L. vannamei* shrimp suffering from WSD.
