# Supplementary material for: The Way of Water: Unravelling White Spot Syndrome Virus (WSSV) Transmission Dynamics in Litopenaeus vannamei Shrimp
Source: Viruses. 2023 Aug 28;15(9):1824. doi: 10.3390/v15091824 (PMC10534367; doi:10.3390/v15091824)
Supplement: Supplementary file 1 [file viruses-15-01824-s001.zip › viruses-2558001-supplementary.pdf]

viruses

Supplementary Materials

The way of water: unravelling white spot syndrome virus (WSSV) transmission dynamics in *Litopenaeus vannamei* shrimp

Natasja Cox [1,2*], Evelien De Swaef[1], Mathias Corteel[1], Wim Van Den Broeck[4], Peter Bossier[3], João J. Dantas-Lima[1], and Hans J. Nauwynck[2]

* Correspondence: natasja.cox@imaqua.eu or natasja.cox@ugent.be (C.N.)

Citation: To be added by editorial staff during production.

Academic Editor: Firstname Lastname

Received: date
Revised: date
Accepted: date
Published: date

Copyright: © 2023 by the authors. Submitted for possible open access publication under the terms and conditions of the Creative Commons Attribution (CC BY) license (https://creativecommons.org/licenses/by/4.0/).

Table S1. Infectious titers (SID$_{50}$ g^{-1}) of two solid inoculum stocks through i.m. injection of *Litopenaeus vannamei* postlarvae. The experiments were terminated when no mortalities occurred for 48 hours.

Stock 1	Dilution	N	Dead animals	Infectious titer (SID$_{50}$ g^{-1})	$\bar{x}$ infectious titer (SID$_{50}$ g^{-1})
**Replicate 1 (PL75, 2.4g)**	10^{-3}	5	5/5	$10^{7.3}$	$10^{7.7}$
	10^{-4}	5	5/5		
	10^{-5}	5	1/5		
	10^{-6}	5	0/5		
	10^{-7}	5	0/5		
**Replicate 2 (PL73, 1.5g)**	10^{-3}	5	5/5	$10^{7.9}$	
	10^{-4}	5	5/5		
	10^{-5}	5	3/5		
	10^{-6}	5	0/5		
	10^{-7}	5	0/5		
**Replicate 3 (PL69, 2.0g)**	10^{-3}	5	5/5	$10^{7.9}$	
	10^{-4}	5	5/5		
	10^{-5}	5	3/5		
	10^{-6}	5	0/5		
	10^{-7}	5	0/5		

Stock 2	Dilution	N	Dead animals	Infectious titer (SID$_{50}$ g^{-1})	$\bar{x}$ infectious titer (SID$_{50}$ g^{-1})
**Replicate 1 (PL80, 7.1g)**	10^{-3}	5	5/5	$10^{8.8}$	$10^{8.6}$
	10^{-4}	5	5/5		
	10^{-5}	5	5/5		
	10^{-6}	5	4/5		
	10^{-7}	5	1/5		
**Replicate 2 (PL80, 7.1g)**	10^{-3}	5	5/5	$10^{8.5}$	
	10^{-4}	5	5/5		
	10^{-5}	5	5/5		
	10^{-6}	5	3/5		
	10^{-7}	5	0/5		
**Replicate 3 (PL80, 7.1g)**	10^{-3}	5	5/5	$10^{8.5}$	
	10^{-4}	5	5/5		
	10^{-5}	5	5/5		
	10^{-6}	5	3/5		
	10^{-7}	5	0/5		

Table S2. Concentration of *vp19* in water samples taken at 0, 24, 30, 48, 54, and 72hpi from tanks housing a *L. vannamei* shrimp suffering from WSD. Shrimp were removed from the tank shortly after their death. ND: Not detected.

Shrimp	Onset of anorexia (hpi)	Time of death (hpi)	Sample time (hpi)	*vp19* copy number/mL
**1**	24	54	0	ND
			24	ND
			30	8.93x10^{6}
			48	1.38x10^{7}
			54	2.43x10^{7}
			72	7.84x10^{6}
**2**	24	42	0	ND
			24	ND
			30	1.71x10^{7}
			48	5.63x10^{6}
			54	ND
			72	ND
**3**	24	48	0	ND
			24	ND
			30	1.40x10^{7}
			48	2.48x10^{7}
			54	4.83x10^{6}
			72	ND
**4**	24	42	0	ND
			24	1.20x10^{6}
			30	5.93x10^{6}
			48	6.02x10^{7}
			54	5.47x10^{7}
			72	2.40x10^{7}
**5**	24	66	0	ND
			24	1.10x10^{6}
			30	7.66x10^{6}
			48	7.53x10^{6}
			54	1.97x10^{7}
			72	1.87x10^{8}
**6**	24	42	0	ND
			24	ND
			30	2.28x10^{7}
			48	1.38x10^{7}
			54	2.96x10^{6}
			72	ND
**7**	48	60	0	ND
			24	5.52x10^{6}
			30	ND
			48	1.17x10^{7}
			54	1.86x10^{7}
			72	4.62x10^{9}
**8**	24	66	0	ND
			24	6.16x10^{6}
			30	ND

			48	$2.63x10^7$
			54	$7.46x10^7$
			72	$1.05x10^7$
**9**	48	66	0	ND
			24	ND
			30	ND
			48	ND
			54	ND
			72	$9.22x10^6$
**10**	24	48	0	ND
			24	ND
			30	$5.26x10^6$
			48	$6.98x10^7$
			54	$1.56x10^8$
			72	$2.63x10^7$
**11**	24	42	0	ND
			24	$1.31x10^7$
			30	$7.04x10^6$
			48	$1.58x10^7$
			54	$1.47x10^7$
			72	ND
